# Urinary nephrin—a potential marker of early glomerular injury: a systematic review and meta-analysis

**DOI:** 10.1007/s40620-023-01585-0

**Published:** 2023-02-20

**Authors:** Belete Biadgo Mesfine, Danica Vojisavljevic, Ranjna Kapoor, David Watson, Yogavijayan Kandasamy, Donna Rudd

**Affiliations:** 1 College of Public Health, Medical and Veterinary Science, James Cook University, 1 James Cook Drive, Douglas, Townsville, QLD 4811 Australia; 2 College of Medicine and Health Sciences, School of Biomedical and Laboratory Sciences, Department of Clinical Chemistry, University of Gondar, Gondar, Ethiopia; 3 Maternal Fetal Medicine Unit and Department of Obstetrics and Gynaecology, Townsville University Hospital, Townsville, Australia; 4 Townsville University Hospital, 100 Angus Smith Dr, Douglas, QLD 4814 Australia

**Keywords:** Urinary nephrin, Nephrinuria, Glomerular injury

## Abstract

**Background:**

Both early recognition of glomerular injury and diagnosis of renal injury remain important problems in clinical settings, and current diagnostic biomarkers have limitations. The aim of this review was to determine the diagnostic accuracy of urinary nephrin for detecting early glomerular injury.

**Methods:**

A search was conducted through electronic databases for all relevant studies published until January 31, 2022. The methodological quality was evaluated using the Quality Assessment of Diagnostic Accuracy Studies (QUADAS-2) tool. Pooled sensitivity, specificity, and other estimates of diagnostic accuracy were determined using a random effect model. The Summary Receiver Operating Characteristics (SROC) was used to pool the data and to estimate the area under the curve (AUC).

**Results:**

The meta-analysis included 15 studies involving 1587 participants. Overall, the pooled sensitivity of urinary nephrin for detecting glomerular injury was 0.86 (95% CI 0.83–0.89) and specificity was 0.73 (95% CI 0.70–0.76). The AUC-SROC to summarise the diagnostic accuracy was 0.90. As a predictor of preeclampsia, urinary nephrin showed a sensitivity of 0.78 (95% CI 0.71–0.84) and specificity of 0.79 (95% CI 0.75–0.82), and as a predictor of nephropathy the sensitivity was 0.90 (95% CI 0.87–0.93), and specificity was 0.62 (95% CI 0.56–0.67). A subgroup analysis using ELISA as a method of diagnosis showed a sensitivity of 0.89 (95% CI 0.86–0.92), and a specificity of 0.72 (95% CI 0.69–0.75).

**Conclusion:**

Urinary nephrin may be a promising marker for the detection of early glomerular injury. ELISA assays appear to provide reasonable sensitivity and specificity. Once translated into clinical practice, urinary nephrin could provide an important addition to a panel of novel markers to help in the detection of acute and chronic renal injury.

**Graphical abstract:**

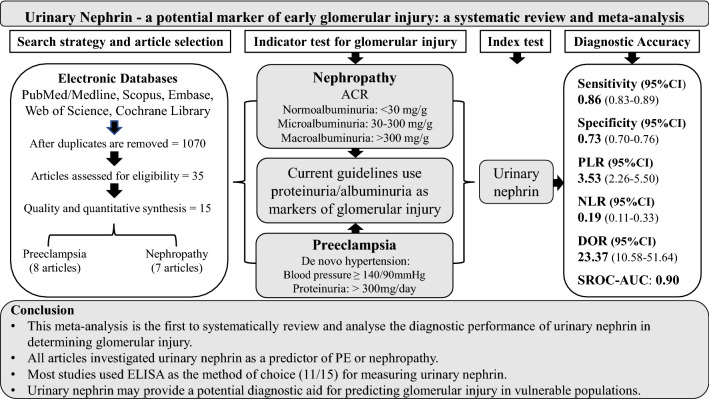

**Supplementary Information:**

The online version contains supplementary material available at 10.1007/s40620-023-01585-0.

## Introduction

Glomerular injury is structural damage to the glomeruli resulting in declining renal function. Glomerular injury, characterised by moderate to severe proteinuria [1], is well established as a prominent contributor to end-stage kidney disease (ESKD) worldwide [2, 3]. Early glomerular injury has been associated with podocyte loss and the development of proteinuria [4] and may also contribute to acute kidney injury (AKI) through progressive damage to nephrons [5]. Repeated glomerular injury and loss of nephron function lead to altered renal perfusion and hyperfiltration, leaving the remaining nephrons at greater risk of injury [6, 7].

Acute kidney injury also puts the kidney at risk of long-term damage. AKI is a clinical term that describes a spectrum of injury events that set the scene for further renal damage [5]. AKI is complex and has a varied aetiology including haemodynamic changes, oxidative stress [8], endothelial damage [9], mitochondrial damage, and immune-mediated mechanisms [8]; about 10% of cases arise from glomerulopathies [10]. A number of studies have investigated novel markers for detecting AKI. The choice of these markers reflects the varied aetiologies of AKI, including but not limited to, kidney injury molecule-1, cell cycle arrest markers tissue inhibitor of metalloproteinase 2; insulin like growth factor binding proteins, neutrophil gelatinase-associated lipocalin and interleukin-18 [11]. These markers are the subjects of numerous excellent reviews and meta-analyses [12, 13]. More recently, attention has turned to investigating the appearance of podocyte proteins in urine following AKI in a number of clinical settings; after surgery and ischaemia reperfusion injury [14, 15]. In order to provide a complete clinical picture of acute and chronic renal damage, the addition of a sensitive indicator of glomerular injury could prove valuable.

Glomerular injury, indicated by the leakage of cells and proteins into the urine [1, 16, 17], is used as a clinical indicator for glomerular damage. There are several well-established biomarkers used for diagnosing and monitoring glomerular damage either alone or in combination [3, 18–21]. However, to date, no biomarker has been identified for early detection of acute glomerular injury [22, 23]. Recent studies have suggested that podocyte proteins may be a better marker for detection of early glomerular injury [24–27]. A number of studies have found nephrin to be a promising early marker of glomerular injury [24, 28–32]. Nephrin, a 180 KD transmembrane protein, is an integral structural component of glomerular podocytes [33]. It belongs to the immunoglobulin superfamily of cell adhesion receptors, and is expressed in glomerular podocytes [33, 34].

The use of urinary nephrin as an indicator of glomerular damage for the prediction of preeclampsia (PE) and glomerular nephritis has been well studied. A nationwide cohort study revealed that there is a strong association between PE and later glomerular injury [35], while another study also showed glomerular injury in diabetic nephropathy [24]. These studies revealed that glomerular injury may occur irrespective of proteinuria, that nephrinuria is often detected prior to proteinuria/albuminuria, and that urine nephrin levels correlate with disease severity [25, 28, 36]. To date, no study has systematically reviewed and analysed the diagnostic accuracy of urinary nephrin for determining glomerular injury in patients with acute and chronic renal injury. This review aims to systematically explore the literature to determine the pooled sensitivity and specificity of urinary nephrin for determining glomerular injury.

## Materials and methods

### Design and protocol registration

This review was performed in accordance with the Preferred Reporting Item for Systematic Review and Meta-analysis Protocol (PRISMA-P 2020) guideline [37]. The review protocol was developed before literature searching and was registered with the International Prospective Register of Systematic Reviews (PROSPERO) database with registration number CRD42022309659.

### Data source and search strategy

This meta-analysis is intended to explore the diagnostic accuracy of urinary nephrin as a biomarker of early glomerular injury. The literature search for eligible studies was performed using electronic databases PubMed/Medline, SCOPUS, EMBASE, Science Direct, Web of Sciences, and Cochrane Database Library of Systematic Reviews from commencement to January 31, 2022. An updated search on August 26, 2022, yielded no additional articles relevant to the topic. We also performed a manual search using Google, and Google Scholar after retrieving articles from the database.

The database was systematically searched in accordance with the Medical Subject Headings Thesaurus (MeSH) and Boolean operators (AND, OR). The key terms used in searching were “glomerular injury” AND “urinary nephrin” OR “nephrinuria”. To capture more articles on early glomerular injury and AKI, additional search key terms were included separately as “Preeclampsia” OR “PE” AND “urinary nephrin” OR “nephrinuria”; “nephropathy” OR “Diabetes nephropathy” AND “urinary nephrin” OR “nephrinuria”; “Acute Kidney Injury” OR “AKI” AND “urinary nephrin” OR “nephrinuria”. The search keywords were searched alone and in all possible combinations with other keywords. Moreover, references from retrieved articles were also reviewed to identify cited articles not captured by electronic database searches.

### Study selection

Original articles that explored the performance of urinary nephrin in the diagnosis of glomerular injury were included. The authors used the EndNote X9 (Thomson Reuters, New York, USA) bibliography manager to check the title and abstracts of the articles and then retrieved and rescreened the selected articles. Duplicate articles were removed electronically, and manually if differences in the citation style of the various journals existed. The reference lists of the eligible articles were checked to find additional relevant articles.

The inclusion and exclusion criteria were systematically applied to studies before they were included in the meta-analysis. Studies eligible for meta-analysis included those that measured urinary nephrin, and studies reporting mandatory data from which the diagnostic accuracy of urinary nephrin could be calculated and which used a reference standard test to classify glomerular injury based on the standardised guidelines. In this regard, PE was classified according to the American Congress of Obstetrics and Gynaecology (ACOG) definition, while diabetic nephropathies were defined according to Kidney Disease Improving Global Outcome (KDIGO) guidelines based on measurements of urine albumin to creatinine ratio (ACR) as; normoalbuminuria (ACR < 30 mg/g), microalbuminuria (ACR = 30–300 mg/g), macroalbuminuria (ACR > 300 mg/g) groups. Only articles published in English were taken into consideration. Studies with duplicate data, review articles, articles which failed to report necessary information, letters to the editor, short communications and conference proceedings were excluded. Initially, two authors (BM and DR) independently reviewed the titles and abstracts of all articles to evaluate the eligibility of the articles. For the studies that could not be judged through the abstracts and titles, the full texts of the original articles were retrieved for detailed evaluation.

### Outcomes of interest

The main outcome of interest of this meta-analysis was the pooled diagnostic accuracy of urinary nephrin (diagnostic sensitivity, specificity, and other estimates of diagnostic accuracy) for determining glomerular injury. Subgroup analysis was also performed to determine the diagnostic accuracy of urinary nephrin according to clinical conditions, the commonly used assay methodology, study design and reported units.

### Data extraction and quality assessment

Data were extracted for all eligible studies. The basic characteristics of the studies were collected using a Microsoft Excel data extraction form, and included the name of the first author, year of publication, country, study design, sample size, clinical condition, method of analysis, reference test, reported cut-off values of urinary nephrin, performance of the test including true positives (TP), true negatives (TN), false positives (FP), and false negatives (FN), if applicable in the studies. If the studies did not report the mandatory outcome data, the 2 × 2 table was extracted from the study to calculate the TP/TN/FP/FN values.

The methodological quality of the studies was evaluated using the Quality Assessment of Diagnostic Accuracy Studies (QUADAS-2) tool [38], which is an improved, redesigned, widely accepted, and validated tool to evaluate the source of bias and variation in diagnostic accuracy studies in systematic reviews. The tool includes four key realms such as patient selection, index test, reference standard, and flow of patients through the study and timing index test and reference standard. Each domain was assessed for the risk of bias and applicability and classified as “low risk of bias”, and “low concern” was considered as having high methodological quality. Any discrepancies in the study selection, data extraction, and/or quality assessment were resolved by discussion with other authors to reach a final consensus. The QUADAS-2 tool scoring criteria was modified according to our aim (Supplementary Table 1).

### Data synthesis and statistical analysis

The data were entered into Microsoft Excel and exported to Meta-Disc version 1.4 software (Complutense University of Madrid, Spain) [39] for analysis. The discriminatory power of a diagnostic test is commonly assessed by measuring how well it correctly identifies true positive and true negative test results in terms of sensitivity and specificity [40]. Pooled sensitivities and specificities, positive likelihood ratio (LR), negative LR, and diagnostic odds ratio (DOR) with a 95% confidence interval (CI) were obtained using the random-effect model (Dersimonian Laird methods) depending on the heterogeneity of the study group. Forest plots of sensitivities, specificities, positive LR, negative LR, and DOR were presented. Furthermore, area under the curve-summary receiver operating characteristics (AUC-SROC) values with 95% CI and Cochrane indices (Q) were calculated. The AUC-SROC was calculated, and the value was defined according to the guideline recommended by Swets in 1988 [41] as; excellent diagnostic accuracy AUC: 0.9–1.0, very good AUC: 0.8–0.9, good AUC: 0.7–0.8 and sufficient diagnostic accuracy with AUC: 0.6–0.7.

The magnitude of inter-study heterogeneity was assessed using visual inspection of the forest plots of accuracy estimates. If no heterogeneity is present, the estimates from individual studies lie along a line corresponding to the pooled accuracy estimate, a large deviation from the pooled estimate indicates possible heterogeneity [39]. Furthermore, statistically measured by the Cochrane *Q* test, a significant *Q* test (*P* < 0.05) suggests the presence of heterogeneity. Further assessment was carried out using the inconsistency index of heterogeneity (*I*^2^ statistics) values of 25%, 50%, and 75% indicated to represent low, medium, and high heterogeneity, respectively [42]. To further assess the heterogeneity subgroup, analyses were conducted based on different parameters including clinical conditions, diagnostic methods, study designs and reported units.

The threshold effect was evaluated by constructing the SROC to assess for presence of shoulder arm pattern for each data point in the plot. A typical shoulder arm pattern indicates the presence of a threshold effect. Further assessment of the threshold effect was conducted and indicated by the presence of a strong positive correlation using a computation of Spearman’s correlation coefficient (*r*^2^) between the logit of sensitivity and logit of 1-specificity [39].

## Results

**Fig. 1:**
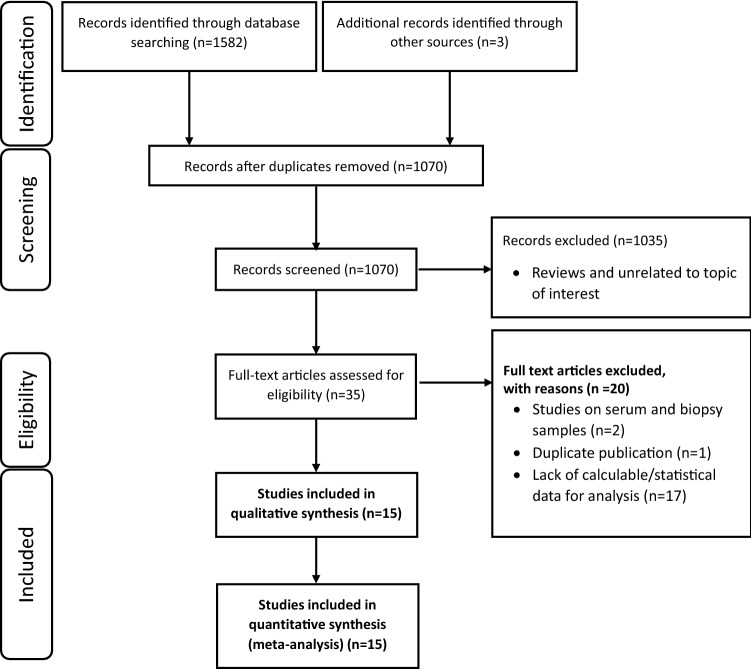
The PRISMA flow diagram illustrating the process of studies reviewed, screened, and included in a systematic review and meta-analysis

Overall, the initial search identified 1585 relevant articles through various database searches, of which 515 were excluded because of duplication. Of the remaining 1070 studies, 1035 were excluded after screening the titles and abstracts, as the articles are not relevant to the current review. Of these, 35 full-text articles were assessed for eligibility. After screening the full texts for calculable statistics, 15 studies that included 1587 participants were included in the meta-analysis. All included studies were published between 2011 and January 2022. Flow diagram illustrating the process of the literature screening method is described in Fig. 1.

### Characteristics of studies included for review

An analysis of the 15 selected studies revealed that all studies aimed to investigate urinary nephrin as an early indicator of glomerular damage in both acute and chronic conditions. Eight studies utilised urinary nephrin for predicting PE [20, 25, 27, 31, 32, 43–45], six studies utilised urinary nephrin for predicting nephropathy [24, 36, 46–49], and one study utilised urinary nephrin for predicting glomerulopathy/glomerular injury [50]. Most of the studies used Enzyme Linked Immunosorbent Assay (ELISA) (*n* = 11) and the remaining used Real Time-Polymerase Chain Reaction (RT-PCR) (*n* = 3), and Western Blotting (WB) (*n* = 1) to determine the concentration of urinary nephrin. All studies reported urinary ACR and/or hypertension with proteinuria (> 300 mg/day) as a reference indicator for glomerular injury. Most articles included in this review report urinary nephrin in two ways: urinary nephrin concentration (*n* = 10) with reported cut-off values for nephrin concentration ranging from 85 to 850 ng/ml, and urinary nephrin corrected by urinary creatinine concentration and reported as urinary nephrin to creatinine ratio (NCR) (*n* = 5), with reported cut-off values ranging from 86.6 to 622 ng/mg. Prospective cohort studies made up 47% of the included studies. The basic characteristics of the eligible studies are summarised in Table 1.

**Table 1:** Characteristics of studies included in the meta-analysis of urinary nephrin to determine early glomerular injury

Author’s name (year)	Country	Study design	Clinical conditions	Sample size	Index test	Methods	Reference test	Cut-off (nephrin)
Jim et al. (2014) [20]	USA	Cohort	Preeclampsia	91	NCR	ELISA	ACOG guideline	≥ 100 ng/mg
Yang et al. (2013) [43]	South Korea	Case–control	Preeclampsia	83	Urine nephrin	ELISA	ACOG guideline	85 ng/ml
Kelder et al. (2012) [44]	Netherlands	Case–control	Preeclampsia	81	Urine nephrin	RT-PCR	ACOG guideline	NR
Son et al. (2011) [27]	South Korea	Case–control	Preeclampsia	45	Urine nephrin	WB	ACOG guideline	NR
Zhai et al. (2016) [32]	Japan	Cohort	Preeclampsia	89	NCR	ELISA	ACOG guideline	122 ng/mg
Zhai et al. (2016) [45]	Japan	Cohort	Preeclampsia	254	NCR	ELISA	ACOG guideline	86.6 ng/mg
Jung et al. (2017) [31]	South Korea	Cohort	Preeclampsia	117	Urine nephrin	ELISA	ACOG guideline	850 ng/ml
Kostovska et al. (2021) [25]	North Macedonia	Cross-sectional	Preeclampsia	101	Urine nephrin	ELISA	ACOG guideline	304.6 ng/ml
Kishore et al. (2021) [36]	India	Cross-sectional	Nephropathy	170	Urine nephrin	ELISA	ACR JCDNP guideline	97.5 ng/ml
Kostovska. (2020) [24]	North Macedonia	Cross-sectional	Nephropathy	120	Urine nephrin	ELISA	ACR KDIGO guideline	255 ng/ml
Heimlich et al. (2018) [50]	Malawi	Cross-sectional	Glomerulopathy	101	NCR	ELISA	ACR KDIGO guideline	622 ng/mg
doNascimento et al. (2013) [46]	Brazil	Cohort	Nephropathy	101	Urine nephrin	RT-PCR	ACR KDIGO guideline	NR
Fayed et al. (2019) [47]	Egypt	Cohort	Nephropathy	80	Urine nephrin	RT-PCR	ACR KDIGO guideline	≥ 3.30
Shahid et al. (2017) [48]	Pakistan	Cohort	Nephropathy	78	Urine nephrin	ELISA	ACR KDIGO guideline	NR
Jim et al. (2012) [49]	USA	Cross-sectional	Nephropathy	76	NCR	ELISA	ACR KDIGO guideline	≥ 100 ng/mg

*ACR* Albumin to Creatinine Ratio, *ELISA* Enzyme Linked Immunosorbent Assay, *RT-PCR* Real Time Polymerase Chain Reaction, *NCR* Nephrin to Creatinine Ratio, *WB* Western Blotting, *ACOG* American Congress of Obstetrics and Gynaecology, *JCDNP* Joint Committee of Diabetes Nephropathy, *NR* Not Reported, *KDIGO* Kidney Disease Improvement Global Outcome, *I*^2^ Inconsistency Index

The quality and risk of bias of the studies were assessed using the QUADAS2 tool [38]. Overall, the studies included in this review were found to be of good quality. While there was a low risk of bias observed in the studies, some studies, such as those by Kelder et al. 2012 [44], Son et al. 2011 [27], do Nascimento et al. 2013 [46], and Shahid et al. 2017 [48] did not provide information on the index test interpretation and did not provide the cut-off value used to interpret the test. Other studies by Yang et al. 2013 [43], Kelder et al. 2012 [44], and Son et al. 2011 [27] introduced bias during patient selection (case–control studies) and reported insufficient data to judge the quality based on the criteria. The modified QUADAS-2 quality appraisal criteria checklist and scoring and percentages of each risk category are presented in Supplementary Table 1 and Supplementary Fig. 1, respectively.

### Subgroup analysis based on assay methodology

Subgroup analysis showed that a difference in the measurement of urinary nephrin was observed based on the assay methodology. ELISA showed a pooled sensitivity of 0.89 (95% CI 0.86–0.92, *I*^2^ = 71.9%) and pooled specificity of 0.72 (95% CI 0.69–0.75, *I*^2^ = 92.7%). The pooled positive LR was 3.84 (95% CI 2.23–6.63), negative LR was 0.16 (95% CI 0.08–0.30), and pooled DOR was 31.55 (95% CI 12.12–82.14). Urinary nephrin using ELISA showed excellent diagnostic accuracy with an AUC of 0.92 (Table 2). Diagnostic accuracy of urinary nephrin observed from three studies using RT-PCR [44, 46, 47] showed a pooled sensitivity of 0.73 (95% CI 0.64–0.81, *I*^2^ = 83.4%) and a pooled specificity of 0.69 (95% CI 0.59–0.79, *I*^2^ = 60.1%) and good diagnostic accuracy AUC of 0.77 (Table 2). Higher diagnostic accuracy was observed in a single study [27] using Western blot analysis with a sensitivity of 0.98 (95% CI 0.83–1.00) and specificity of 0.98 (95% CI 0.80–1.00).

**Table 2:** Subgroup analysis of urinary nephrin as a potential marker of early glomerular injury

Studies	Sensitivity (95% CI)	Specificity (95% CI)	Positive LR (95% CI)	Negative LR (95% CI)	DOR (95%CI)	AUC
All studies	0.86 (0.83–0.89)	0.73 (0.70–0.76)	3.53 (2.26–5.50)	0.19 (0.11–0.33)	23.37 (10.58–51.64)	0.90
*I*^2^ (%)	79.5	90.8	92.8	79.8	73.3	
Clinical condition						
Preeclampsia	0.78 (0.71–0.84)	0.79 (0.75–0.82)	5.35 (2.72–10.52)	0.24 (0.11–0.52)	18.08 (5.11–64.02)	0.91
* I*^2^ (%)	81.4	89.2	85.8	82	78.4	
Nephropathy	0.90 (0.87–0.93)	0.62 (0.56–0.67)	2.49 (1.44–4.30)	0.16 (0.10–0.26)	22.10 (10.43–46.82)	0.90
* I*^2^ (%)	63.7	89.7	94	35.7	41.5	
Study design						
Cohort	0.86 (0.80–0.91)	0.71 (0.66–0.74)	3.04 (1.64–5.64)	0.28 (0.15–0.53)	13.23 (4.43–39.57)	0.87
* I*^2^ (%)	73	94.6	93.5	56.3	68	
Case–Control	0.72 (0.62–0.81)	0.82 (0.74–0.88)	3.70 (1.59–8.57)	0.32 (0.11–0.95)	15.32 (2.41–97.23)	0.97
* I*^2^ (%)	89.9	65.7	63.3	84.5	79.6	
Cross-sectional	0.92 (0.88–0.95)	0.73 (0.67–0.79)	3.74 (2.07–6.76)	0.10 (0.05–0.19)	53.74 (26.09–110.71)	0.95
* I*^2^ (%)	59.4	86.2	84.9	39	28.2	
Diagnostic method						
ELISA	0.89 (0.86–0.92)	0.72 (0.69–0.75)	3.84 (2.23–6.63)	0.16 (0.08–0.30)	31.55 (12.12–82.14)	0.92
* I*^2^ (%)	71.9	92.7	94.6	73.2	72.1	
RT-PCR	0.73 (064–0.81)	0.69 (0.59–0.79)	2.16 (1.56–2.99)	0.39 (0.20–0.75)	6.17 (3.19–11.94)	0.77
* I*^2^ (%)	83.4	60.1	4.2	66.7	0.0	
Nephrin reporting methods						
Urinary nephrin (ng/ml)	0.86 (0.82–0.89)	0.77 (0.72–0.80)	4.66 (2.09–10.41)	0.18 (0.09–0.34)	27.36 (10.45–71.69)	0.92
* I*^2^ (%)	86.6	92.4	95.6	82.8	75.1	
Urinary NCR (ng/mg)	0.89 (0.81–0.94)	0.69 (0.64–0.73)	2.48 (1.68–3.64)	0.19 (0.05–0.67)	17.76 (3.71–85.13)	0.86
* I*^2^ (%)	78.3	84.8	75.7	77.5	73.0	

*AUC* area under the curve, *CI* confidence interval, *DOR* diagnostic odds ratio, *ELISA* enzyme linked immunosorbent assay, *LR* likelihood ratio, *RT-PCR* real time polymerase chain reaction, *NCR* nephrin to creatinine ratio

### Subgroup analysis based on study designs

Seven of the 15 studies used a prospective cohort study design. The pooled sensitivity of urinary nephrin in prospective cohort studies was 0.86 (95% CI 0.80–0.91, *I*^2^ = 73%) and a pooled specificity of 0.71 (95% CI 0.66–0.74, *I*^2^ = 94.6%) and the AUC was 0.87 (Table 2). Overall, the diagnostic accuracy of urinary nephrin in all study designs was very good (AUC 0.8–0.9). However, the high level of heterogeneity following subgroup analysis was still observed across studies.

### Subgroup analysis based on reporting units of urinary nephrin

In this review, 10 of the 15 studies did not correct urinary nephrin concentration for the urine creatinine concentration. Measurement of urinary nephrin (ng/ml) showed a pooled sensitivity of 0.86 (95% CI 0.82–0.89) and pooled specificity of 0.77 (95% CI 0.72–0.80). The normalised urinary nephrin by correction with urine creatinine reported as NCR (ng/mg) showed a pooled sensitivity of 0.89 (95% CI 0.81–0.94) and specificity of 0.69 (95% CI 0.64–0.73) (Table 2).

### Overall diagnostic accuracy of urinary nephrin

**Fig. 2:**
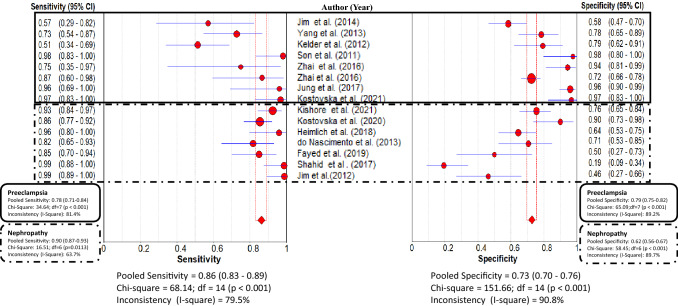
Forest plot of the pooled sensitivity and specificity of urinary nephrin for detecting glomerular injury across all studies. Subgroup analysis: Preeclampsia (within solid lines) and Nephropathy (within dashed lines) shows the pooled sensitivity and specificity of urinary nephrin for detecting these conditions. The circles and the horizontal lines represent the point estimate and 95% CI, respectively. Between the dotted vertical lines represents the pooled estimate, and the diamonds represent the pooled estimate in all studies with 95% CI

**Fig. 3:**
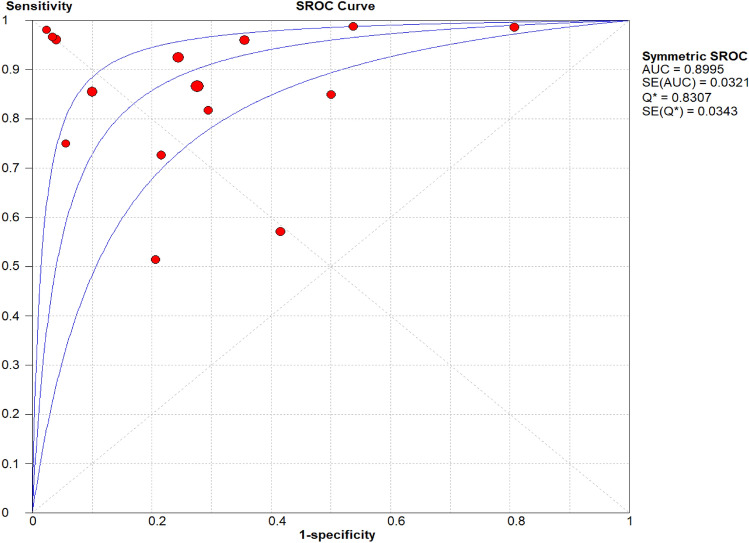
Hierarchical Summary Receiver Operating Characteristics (SROC) plot of urinary nephrin to determine glomerular injury across all settings. The SROC curve is represented by the middle line; each of the analysed studies is represented by a circle and the respective 95% CI, by the two upper and lower lines

The pooled sensitivity of urinary nephrin for detecting glomerular injury was 0.86 (95% CI 0.83–0.89) and the pooled specificity was 0.73 (95% CI 0.70–0.76) (Fig. 2). The AUC obtained from the SROC was 0.90 (Fig. 3). This result suggests that urinary nephrin achieved high diagnostic accuracy in diagnosing glomerular injury due to the observation that AUC > 0.7 is a risk predictor. The pooled positive LR was 3.53 (95% CI 2.26–5.50) and negative LR was 0.19 (95% CI 0.11–0.33). Moreover, using a random-effect model, the DOR was 23.37 (95% CI 10.58–51.64) (Table 2).

No significant heterogeneity was found between the studies based on the threshold effect (*r*^2^) = − 0.17; *P* = 0.57. A non-threshold effect using estimation of Chi-square test (*P* < 0.05) and inconsistency index for pooled sensitivity (*I*^2^ = 79.5%) and specificity (*I*^2^ = 90.8%) indicated that there was heterogeneity in the value of urinary nephrin among the 15 studies (Fig. 2) due to differences between the diagnostic methods, clinical conditions, cut-off values, and study designs of the studies included in the review. Therefore, subgroup analysis was conducted based on the clinical conditions, assay methodology, study designs and units used for reporting urinary nephrin**.**

### Subgroup analysis based on clinical conditions

Subgroup analysis based on clinical conditions showed that urinary nephrin predicts glomerular injury caused by PE with a pooled sensitivity of 0.78 (95% CI 0.71–0.84, *I*^2^ = 81.4%) and a pooled specificity of 0.79 (95% CI 0.75–0.82, *I*^2^ = 89.2%) (Fig. 2). Furthermore, urinary nephrin predicts glomerular injury caused by nephropathy with a pooled sensitivity of 0.90 (95% CI 0.87–0.93, *I*^2^ = 63.7%), and specificity of 0.62 (95% CI 0.56–0.67, *I*^2^ = 89.7%) (Fig. 2). Urinary nephrin shows excellent diagnostic accuracy for predicting glomerular injury caused by either PE (with AUC of SROC 0.91) or nephropathy with AUC of the SROC 0.90 for predicting nephropathy (Table 2).

### Sensitivity analysis

Sensitivity analysis was conducted to assess the impact of each study in the interpretation of the diagnostic accuracy of urinary nephrin on the overall diagnostic accuracy. The sensitivity analysis performed to check heterogeneity was conducted by excluding each study step by step from the analysis. The estimate showed that the excluded study did not lead to significant changes in the overall AUC of the index test (urinary nephrin) (Table 3).

**Table 3:** Sensitivity analysis (pooled diagnostic accuracy with 95% CI, when the individual study was excluded from the meta-analysis)

First author (year)	Sensitivity (95% CI)	Specificity (95% CI)	Positive LR (95% CI)	Negative LR (95% CI)	DOR (95% CI)	AUC
All studies	0.86 (0.83–0.89)	0.73 (0.70–0.76)	3.53 (2.26–5.50)	0.19 (0.11–0.33)	23.37 (10.58–51.64)	0.90
Jim et al. (2014) [20]	0.87 (0.84–0.90)	0.74 (0.71–0.77)	3.86 (2.39–6.24)	0.16 (0.09–0.29)	27.89 (13.09–59.44)	0.91
Yang et al. (2013) [43]	0.87 (0.84–0.90)	0.72 (0.69–0.75)	3.56 (2.23–5.70)	0.17 (0.09–0.32)	26.46 (10.98–63.74)	0.91
Kelder et al. (2012) [44]	0.89 (0.86–0.92)	0.72 (0.69–0.75)	3.65 (2.28–5.84)	0.70 (0.10–0.29)	27.61 (12.06–63.21)	0.92
Son et al. (2011) [27]	0.86 (0.82–0.89)	0.72 (0.69–0.75)	3.32 (2.16–5.10)	0.20 (0.12–0.35)	19.96 (9.28–42.92)	0.89
Zhai et al. (2016) [32]	0.86 (0.83–0.89)	0.72 (0.69–0.75)	3.29 (2.11–5.14)	0.18 (0.10–0.33)	22.42 (9.81–51.23)	0.89
Zhai et al. (2016) [45]	0.86 (0.83–0.89)	0.73 (0.69–0.76)	3.67 (2.22–6.07)	0.19 (0.10–0.33)	24.57 (7.98–42.30)	0.90
Jung et al. (2017) [31]	0.86 (0.83–0.89)	0.70 (0.67–0.73)	3.07 (2.00–4.70)	0.20 (0.11–0.34)	19.84 (9.14–43.09)	0.88
Kishore et al. (2021) [36]	0.85 (0.82–0.88)	0.72 (0.69–0.75)	3.52 (2.20–5.63)	0.20 (0.12–0.36)	22.75 (9.67–53.53)	0.90
Kostovska et al. (2020) [24]	0.86 (0.83–0.90)	0.72 (0.69–0.75)	3.29 (2.13–5.09)	0.18 (0.10–0.34)	21.85 (9.51–50.21)	0.90
Heimlich et al. (2018) [50]	0.86 (0.82–0.89)	0.73 (0.70–0.76)	3.71 (2.25–6.12)	0.20 (0.11–0.35)	22.62 (9.88–51.78)	0.90
Do Nascimento et al. (2013) [46]	0.87 (0.83–0.90)	0.73 (0.70–0.76)	3.63 (2.26–5.85)	0.18 (0.10–0.33)	25.99 (10.87–62.12)	0.91
Fayed et al. (2019) [47]	0.86 (0.83–0.89)	0.73 (0.70–0.76)	3.82 (2.35–6.23)	0.18 (0.10–0.32)	27.00 (11.57–63.01)	0.91
Shahid et al. (2017) [48]	0.85 (0.82–0.88)	0.75 (0.72–0.78)	3.51 (2.49–4.94)	0.19 (0.11–0.34)	23.98 (10.52–40.47)	0.90
Kostovska et al. (2021) [25]	0.86 (0.82–0.89)	0.72 (0.69–0.75)	3.24 (2.11–4.96)	0.21 (0.12–0.36)	19.06 (8.92–40.71)	0.88
Jim et al. (2012) [49]	0.85 (0.82–0.88)	0.74 (0.70–0.76)	3.84 (2.33–6.33)	0.20 (0.12–0.35)	22.30 (9.88–50.36)	0.90

*AUC* area under the curve, *CI* confidence interval, *DOR* diagnostic odds ratio, *LR* likelihood ratio

## Discussion

This review shows that urine nephrin could be a potential indicator of early glomerular injury, as demonstrated by a very good diagnostic accuracy in patients with acute and chronic renal injury. Indeed, urinary nephrin has demonstrated potential as a marker for early glomerular injury in several studies [29, 30, 32] and could prove to be a useful routine diagnostic marker used alone or in combination with other novel markers such as neutrophil gelatinase-associated lipocalin and cell cycle arrest markers [11, 51, 52] for the prediction of early kidney injury. However, appropriate validation of new diagnostic biomarkers requires the demonstration of assay performance against validation and verification criteria set out by professional organisations [53]. Progression into clinical use requires an investigation of the diagnostic accuracy and the ability of the assay to discriminate between diseased and healthy populations. In this regard, our review aims to provide a first step in this process.

Despite proven satisfactory diagnostic accuracy of urinary nephrin (AUC-SROC 0.9), heterogeneity exists across the studies reviewed; this has been documented previously [54] and across other studies [21, 26, 55]. The potential source of heterogeneity in this review was evaluated using subgroup analysis by clinical condition, methods of analysis, study design, and reporting units. The analysis also showed heterogeneity existed within subgroups, nevertheless, the diagnostic accuracy of urinary nephrin was considered satisfactory in each group following subgroup analysis.

Laboratories often have to find a balance between diagnostic accuracy and technical complexity when choosing assays to adopt for routine diagnostic use [56]. Therefore, an important aspect to include in a meta-analysis such as this is the heterogeneity in diagnostic accuracy of the methods employed by the various studies. ELISA was the method of choice in most studies (*n* = 11) for the detection of nephrinuria in PE and nephropathies [20, 24, 25, 31, 32, 36, 43, 45, 48–50]. Interestingly these assays demonstrated improved sensitivity (0.89) and specificity (0.73) and therefore diagnostic accuracy (AUC-SROC = 0.92) for determining urinary nephrin compared to RT-PCR [44, 46, 47]. Additionally, one study demonstrated a sensitivity and specificity of 100% in a single centre trial involving 25 women with PE, using Western blot analysis [27].

Under normal physiological conditions, random urine collections contain varied concentrations of urine biomarkers due to variability in urine volume. Therefore, biomarker concentration is often corrected using urinary creatinine [57]. Our result found the pooled sensitivity and specificity of uncorrected urinary nephrin (ng/ml) was 0.86 and 0.77, respectively. In comparison, urinary nephrin normalised by correction for urinary creatinine, NCR (ng/mg), showed a greater pooled sensitivity of 0.89 and lower specificity 0.69. The difference in reporting methods and lack of consistent cut-off values for urinary nephrin may account for heterogeneity seen across the studies included in this review. Hence, a uniform reporting approach for urinary nephrin is mandatory for ease of interpretation and comparison of results across the literature.

The pooled analysis of the studies investigating urinary nephrin as a diagnostic marker of glomerular injury showed good sensitivity and specificity. Studies investigating urinary nephrin predominantly focused on early detection of PE and diabetic nephropathy. Both conditions rely on the detection of albumin or protein in the urine as an indicator of glomerular damage. PE has an acute presentation associated with endothelial swelling and derangements [17] and has also been associated with podocyte loss and nephrin shedding [17]. As is the case with patients suffering AKI, these patients do not always go on to incur further renal impacts and therefore progressive renal decline [58]. Conversely, nephropathy develops over time and could be considered an example of the chronic progression of renal disease [59].

All studies determining urinary nephrin showed that it increased significantly in patients with increased levels of albumin in urine [24, 36]. Likewise, other studies have also shown that urinary nephrin increased linearly with the progression of the disease, this suggests that quantification of nephrin could be a useful biomarker of glomerular injury progression [25, 47, 49].

Overall, the sensitivity and specificity of the individual studies reviewed for predicting glomerular injury of PE ranged from 51–97% to 58–97%, respectively. Urinary nephrin predicts acute glomerular injury caused by PE with a high level of sensitivity (0.78) and specificity (0.79) with SROC of 0.91. Thus, urinary nephrin could be considered a good predictor of disease, showing an improvement in diagnostic accuracy of albumin (ACR) with sensitivity of 36% [20] and protein to creatinine ratio with sensitivity of 72% in predicting significant proteinuria [60].

There is growing evidence that urinary nephrin may be a superior marker for PE and can achieve better diagnostic accuracy than other podocyte biomarkers. Kerley et al. reported improved diagnostic accuracy of urinary nephrin with sensitivity of 0.81 (95% CI 0.72–0.88) and specificity of 0.84 (95% CI 0.79–0.84) when compared to combined podocyte biomarkers [26]. Likewise, a previous meta-analysis by Wu et al. investigating the value of biomarkers for the detection of early-stage PE found a low predictive value using single biomarkers (disintegrin and metalloprotease 12, inhibin-A, pregnancy-associated plasma protein A, placental growth factor and placental protein 13) with a pooled sensitivity of all single biomarkers of 0.40 (95% CI 0.39–0.41) and a pooled specificity of 0.90 (95% CI 0.90–0.90) in 147 studies of 401 laboratory biomarkers [21]. The investigators found increased diagnostic sensitivity and specificity with the use of a panel of biomarkers combined with clinical characteristics; sensitivity of 0.43 (95% CI 0.41–0.46) and specificity of 0.91 (95% CI 0.90–0.91). However, the review by Wu et al. was not focused on glomerular-specific biomarkers for determining glomerular injury. A similar systematic review conducted by the World Health Organisation (WHO) in 2004 assessed the usefulness of combined clinical biophysical and biochemical tests for the prediction of PE [55], concluding that there was yet to be a cost-effective or reliable screening test. It has since been demonstrated that urinary nephrin could possibly fill that role. The improved diagnostic accuracy demonstrated by urinary nephrin may warrant its inclusion in these panels to improve early detection of PE.

Identifying nephropathy in the early stages of the disease prior to proteinuria is challenging. Existing guidelines rely on albuminuria as an indicator of glomerular nephropathy [61]. However, this has limitations in terms of timing for detection of early nephropathy since glomerular structural damage precedes microalbuminuria [30]. In terms of the specificity, ACR is widely accepted for the classification of glomerular injury and chronic kidney disease, andwhile albuminuria has been independently and strongly associated with progression to ESKD [62]. The included studies showed that nephrinuria positively correlated with increases in urinary concentrations of albumin and hyperglycaemia status. However, nephrinuria was also detected in a high proportion of diabetic patients with normoalbuminuria, therefore given that over time hyperglycaemia is likely to further damage renal vasculature and the glomerular filtration barrier, nephrinuria may provide an early indicator of renal damage. Although not all diabetic patients with nephrinuria progress to kidney disease, nephrinuria can be used both as an early indicator of glomerular damage prior to progression to fulminant kidney disease/injury and to signal the need for interventional strategies in this vulnerable population. In this meta-analysis, the diagnostic accuracy of urinary nephrin for predicting glomerular nephropathy showed good diagnostic sensitivity of 0.90 (95% CI 0.87–0.93) and specificity of 0.62 (95% CI 0.56–0.67), SROC = 0.90, suggesting that urinary nephrin may be a promising biomarker of glomerular injury.

Early detection of urinary nephrin before the appearance of protein and albumin in urine could allow for the detection of glomerular injury before the loss of renal function [24]. This is important for early diagnosis and intervention. Furthermore, albuminuria may not always be present; a study by An et al. demonstrated that more than 30% of patients with kidney disease had undetectable albuminuria despite the presence of severe glomerular damage/renal insufficiency [63]. Likewise, previous studies have indicated that podocyte proteins may provide earlier indicators for glomerular nephropathies preceding albuminuria [28, 48, 50, 64]. Studies included in this review detected nephrinuria prior to the presence of albuminuria, and the urinary nephrin concentration reflects the severity of the disease [32, 36]. This has also been reported in previous studies that found nephrinuria was detected prior to albuminuria during glomerular injury. One study showed that 54% of diabetes mellitus patients with normoalbuminuria had nephrinuria and 100% of diabetes mellitus patients with micro-macroalbuminuria had nephrinuria [49]. Similarly, another study demonstrated the presence of elevated nephrinuria in 82% of patients with normoalbuminuria, in 88% of patients with microalbuminuria, and in 100% of patients with macroalbuminuria [24].

The intention of this review was to investigate the role of urinary nephrin as a marker of early glomerular injury for detecting both acute and chronic kidney injury. All studies related to nephropathy demonstrated that urinary nephrin increased in parallel with albuminuria and correlated with the progression of the severity of nephropathy [24, 36, 46–50], suggesting that nephrinuria is a sensitive indicator for nephropathy. It has been suggested that continued attempts at regeneration and upregulation of nephrin production may be evidence of podocyte repair following injury [65]. Urinary nephrin also negatively correlated with the glomerular filtration rate, and increasing levels were associated with the progression of injury to other forms of kidney injury/disease [36, 47].

The diagnostic accuracy of urinary nephrin for detecting PE and diabetic nephropathy could therefore be extrapolated into use as a potential predictor of early glomerular injury, particularly in the setting of AKI. Recently, studies have emerged investigating the value of urinary nephrin for predicting AKI, particularly in critically ill neonates [66, 67]. These studies concluded that urinary nephrin may well provide a marker for predicting AKI, demonstrating a diagnostic sensitivity of 62.5%, 61.5%, and specificity of 82.1%, 76.9% respectively at a cut-off point of NCR = 0.375 µg/mg, suggesting that urinary nephrin may give an early indication of podocyte damage as an indicator of those infants at risk of developing AKI. This is an area of intense interest in the literature [30, 66, 67], since a single biomarker may not suffice to define AKI given inherent renal heterogeneity and the disparate settings under which kidney injury occurs [68].

The strength of this meta-analysis is that it is the first to systematically analyse the pooled diagnostic accuracy of urinary nephrin in the diagnosis of glomerular injury. However, the limitations of this meta-analysis cannot be ignored. First, urine ACR and de novo hypertension were used as a reference standard to stratify cases and controls and to determine the diagnostic accuracy of urinary nephrin as a useful marker for glomerular injury. Second, there was high heterogeneity across the included studies in the meta-analysis. Third, diagnostic cut-off values of urinary nephrin of numerous studies were not consistent and the included articles used different methods of assay measurement. Fourth, the current guidelines for stratifying nephropathies using urine ACR as a reference standard test cannot reveal subclinical glomerular damage and might underscore the specificity of urinary nephrin. Fifth, the majority of studies included in this review are cross-sectional studies, hence, the cross-sectional nature of the study design reflects association rather than causality. While nephrin has been demonstrated to play an important role in the slit diaphragm of the glomerulus providing structural stability [69], normal functioning and repair of damaged glomerulus in acute injury [70–73], there is no supportive evidence for  nephrin as a causal mechanism of glomerular injury. Nor is there evidence to support the early detection of nephrinuria as a reliable predictor of consequent glomerular injury and further progression to other forms of kidney injury/disease in the vulnerable populations.

## Conclusion

Overall, this meta-analysis found that urinary nephrin could become an effective and robust biomarker for the early prediction of glomerular injury as well as for monitoring disease. Perhaps adding urinary nephrin as a marker of early glomerular injury to a panel of promising markers for AKI could provide a more complete clinical picture to help determine renal injury and prognosis in the future.

### Supplementary Information

Below is the link to the electronic supplementary material.

Supplementary file1 (DOCX 126 KB)

Supplementary file2 (DOCX 36 KB)

## Data Availability

The authors confirm that the data supporting the findings of this study are available within the article [and/or] its supplementary materials.
