# Supplementary material for: Urinary nephrin—a potential marker of early glomerular injury: a systematic review and meta-analysis
Source: J Nephrol. 2023 Feb 20;37(1):39–51. doi: 10.1007/s40620-023-01585-0 (PMC10920435; doi:10.1007/s40620-023-01585-0)
Supplement: Supplementary file 1 — Supplementary file1 (DOCX 126 KB) [file 40620_2023_1585_MOESM1_ESM.docx]

**Article title:** Urinary Nephrin - a potential marker of early glomerular injury: a systematic review and meta-analysis

**Journal name:** Journal of Nephrology

**Authors name:** Belete Biadgo Mesfine, Danica Vojisavljevic, Ranjna Kapoor, David Watson, Yogavijayan Kandasamy, and Donna Rudd

**Corresponding author:** Donna Rudd, Discipline of Biomedicine, James Cook University, Australia, [donna.rudd@jcu.edu.au](mailto:donna.rudd@jcu.edu.au)

**Supplementary Figure 1:** Represents Modified Quality Assessment of Diagnostic Accuracy Studies (QUADAS-2) quality scores for risk of bias and applicability concerns from included studies. (A) percentage (%) of studies with a low, unclear, and high risk of applicability; (B) Percentage (%) of studies with a low, unclear, and high risk of bias.
