# Supplementary material for: Urinary nephrin—a potential marker of early glomerular injury: a systematic review and meta-analysis
Source: J Nephrol. 2023 Feb 20;37(1):39–51. doi: 10.1007/s40620-023-01585-0 (PMC10920435; doi:10.1007/s40620-023-01585-0)
Supplement: Supplementary file 2 — Supplementary file2 (DOCX 36 KB) [file 40620_2023_1585_MOESM2_ESM.docx]

**Article title:** Urinary Nephrin-a potential marker of early glomerular injury: a systematic review and meta-analysis

**Journal name:** Journal of Nephrology

**Authors name:** Belete Biadgo Mesfine, Danica Vojisavljevic, Ranjna Kapoor, David Watson, Yogavijayan Kandasamy, and Donna Rudd

**Corresponding authors:** Donna Rudd, Discipline of Biomedicine, James Cook University, Australia, [donna.rudd@jcu.edu.au](mailto:donna.rudd@jcu.edu.au)

**Supplementary Table 1.** Modified Quality Assessment of Diagnostic Accuracy Studies (QUADAS) scoring results of the included studies to summarise the risk of bias and applicability concerns

| **Studies** | **Modified Quality Assessment of Diagnostic Accuracy (QUADAS) criteria** | | | | | | | | | | | | | |
| --- | --- | --- | --- | --- | --- | --- | --- | --- | --- | --- | --- | --- | --- | --- |
| **First Author (Year)** | **Risk of Bias** | | | | | | | | | | **Risk of Applicability** | | | |
|  | PS | PS | PS | IT | IT | RT | RT | FT | FT | FT | PS | PS | IT | RS |
|  | **1** | **2** | **3** | **4** | **5** | **6** | **7** | **8** | **9** | **10** | **11** | **12** | **13** | **14** |
| Jim et al. (2014)^[20]^ | Y | Y | Y | Y | U | Y | Y | Y | Y | U | Y | Y | Y | Y |
| Yang et al. (2013)^[43]^ | Y | N | Y | Y | U | Y | Y | Y | Y | U | Y | Y | Y | U |
| Kelder et al. (2012)^[44]^ | Y | N | Y | U | N | Y | Y | Y | Y | U | Y | Y | Y | U |
| Son et al. (2011)^[27]^ | Y | N | Y | Y | U | Y | Y | Y | Y | Y | Y | Y | Y | U |
| Zhai et al. (2016)^[32]^ | Y | Y | Y | U | Y | Y | Y | Y | Y | Y | Y | Y | Y | Y |
| Zhai et al. (2016)^[45]^ | Y | Y | Y | Y | Y | Y | Y | Y | Y | Y | Y | Y | Y | Y |
| Jung et al. (2017)^[31]^ | Y | Y | Y | Y | U | Y | Y | Y | Y | Y | Y | Y | N | Y |
| Kishore et al. (2021)^[36]^ | Y | Y | Y | Y | Y | Y | Y | Y | Y | Y | Y | Y | Y | Y |
| Kostovska. (2020)^[24]^ | Y | Y | Y | Y | Y | Y | Y | Y | Y | Y | Y | Y | Y | Y |
| Heimlich et al. (2018)^[50]^ | Y | Y | Y | Y | Y | Y | Y | Y | Y | Y | Y | Y | Y | Y |
| doNascimento et al. (2013)^[46]^ | Y | Y | N | Y | U | Y | Y | Y | Y | U | Y | Y | Y | Y |
| Kostovska et al. (2021)^[25]^ | Y | Y | Y | Y | Y | Y | Y | Y | Y | Y | Y | Y | Y | Y |
| Fayed et al. (2019)^[47]^ | Y | Y | Y | Y | Y | Y | Y | Y | Y | Y | Y | Y | Y | Y |
| Shahid et al. (2017)^[48]^ | Y | Y | N | Y | Y | Y | Y | Y | Y | U | Y | Y | Y | Y |
| Jim et al. (2012)^[49]^ | Y | Y | Y | Y | U | Y | Y | Y | Y | Y | Y | Y | Y | Y |

| Modified QUADAS criteria **CHECKLIST** used for assessing the quality of studies included in the meta-analysis | | | |
| --- | --- | --- | --- |
| - The selection criteria are clearly defined, and a consecutive or random sample of patients enrolled? | Yes | No | Unclear |
| - Was a case-control study design avoided? |  |  |  |
| - Were inclusion and exclusion criteria clearly stated? |  |  |  |
| - Was the diagnosis of glomerular injury made without knowledge of the index test results? |  |  |  |
| - Was the index test result interpreted in a blinded fashion? |  |  |  |
| - Was the study described the reference standard to classify the target condition? |  |  |  |
| - Were the reference standard results interpreted without knowledge of the results of the index test? |  |  |  |
| - Were results of the index and reference test collected on the same patients at the same time? |  |  |  |
| - Did all patients receive the same reference standard? |  |  |  |
| - Were all patients included in the analysis? Or were withdrawals from the study explained? |  |  |  |
| - Were samples collected from patients with a high risk of developing kidney injury |  |  |  |
| - Were patients recruited with symptoms consistent with kidney injury? |  |  |  |
| - Were the methods for testing sufficiently explained? |  |  |  |
| - Were intermediate test results reported? |  |  |  |

Note: PS: Patient selection; IT: Index test; RS: Reference standard; FT: Flow and timing
